# High density of peritumoral lymphatic vessels is a potential prognostic marker of endometrial carcinoma: a clinical immunohistochemical method study

**DOI:** 10.1186/1471-2407-10-131

**Published:** 2010-04-08

**Authors:** Ying Gao, Zi Liu, Fei Gao, Xiao-yu Meng

**Affiliations:** 1 Department of Radiotherapy Oncology, the 1st Affiliated Hospital of Medical College of Xi'an Jiaotong University, Xi'an 710061, China

## Abstract

**Background:**

The lymphatic system is a major route for cancer cell dissemination and also a potential target for antitumor therapy. To investigate whether increased lymphatic vessel density (LVD) is a prognostic factor for nodal metastasis and survival, we studied peritumoral LVD (P-LVD) and intratumoral LVD (I-LVD) in samples from 102 patients with endometrial carcinoma;

**Methods:**

Endometrial carcinoma tissues were analyzed for lymphatic vessels by immunohistochemical staining with an antibody against LYVE-1. Univariate analysis was performed with Kaplan-Meier life-table curves to estimate survival, and was compared using the log rank test. Prognostic models used multivariate Cox regression analysis for multivariate analyses of survival;

**Results:**

Our study showed that P-LVD, but not I-LVD, was significantly correlated with lymph vascular space invasion (LVSI), lymph node metastasis, tumor stage, and CD44 expression in endometrial carcinoma. Moreover, P-LVD was an independent prognostic factor for progression-free survival and overall survival of endometrial carcinoma;

**Conclusions:**

P-LVD may serve as a prognostic factor for endometrial carcinoma. The peritumoral lymphatics might play an important role in lymphatic vessel metastasis.

## Background

Endometrial cancer is the most frequent gynaecologic genital malignancy in the western world [1,2] and the five-year overall survival rate for patients with advanced stage cancer is about 65% [3]. Traditional prognostic factors for the disease are histological type, grade, tumor stage, and depth of myometrial invasion. However, even for the patients in the same stage the clinical courses are highly variable. The current diagnostic technology is insufficient to identify endometrial cancer patients with poor prognosis. Since dissemination through lymphatic vessels is the main means of tumor spread, we hypothesized that lymphatic vessel density (LVD) might serve as a prognostic marker for lymph node metastasis and survival.

Lymphangiogenesis has been difficult to study because of the lack of specific lymphatic markers. Recently, this situation has changed with the discovery of lymphangiogenic markers, such as VEGF-C, VEGF-D, VEGFR-3, LYVE-1, PROX1, and podoplanin. Among them, LYVE-1 is a reliable specific marker for lymphatics [4,5]. It is a surface endocytic receptor for hyaluronan [6], which shares 41% homology with the metastasis related CD44 molecule [7]. CD44 binds to hyaluronic acid (HA), major components of the extracellular matix (ECM) and CD44 is important in tumor progression and metastasis [8]. In common with CD44, the LYVE-1 molecule binds both soluble and immobilized HA. HA continuously transits through the lymphatic system and is potentially involved in lymph node homing by CD44^+ ^leukocytes and tumor cells [9]. However, little is known about the role of LYVE-1 in lymphatic metastasis.

In this study, LYVE-1 staining was used to determine LVD in tissue samples from endometrial carcinoma patients. The findings were analyzed in combination with data regarding lymph node metastasis, lymph vascular space invasion (LVSI), CD44 expression, and other clinicopathological parameters. The potential of intratumoral LVD (I-LVD) and peritumoral LVD (P-LVD) as prognostic factors for lymph node metastasis, progression-free survival and overall survival was investigated.

## Methods

### Materials

One hundred and two endometrial hysterectomy specimens containing endometrial carcinoma tissue were obtained from the pathological archives of the 1st Affiliated Hospital of Medical College of Xi'an Jiaotong University from January 1997 to July 2002. Patients with a disease other than endometrial carcinoma were excluded. The present study was approved by the ethics committee of the 1st Affiliated Hospital of Medical College of Xi'an Jiaotong University. All samples were obtained with medical-ethics approval and all patients gave informed consent. All haematoxylin and eosin-stained slides were re-reviewed by a gynaecological pathologist to confirm the diagnosis, histological grade, histological type, surgical stage and lymphangiosis. Pathological stage and histological type were determined according to 1988 International Federation of Gynecology and Obstetrics (FIGO) criteria. Histological classification was performed according to the World Health Organization (WHO) system in well-differentiated (G1; n = 39), moderately differentiated (G2; n = 32) and poorly differentiated (G3; n = 31) carcinomas. Patients with endometrial carcinoma received radical hysterectomy, salpingo-oophorectomy, or selective pelvic lymphadenectomy, with or without para-aortic lymphadenectomy. Lymph node dissection was generally performed in patients having tumors with deep myometrial invasion and/or high-grade or aggressive histological features. The standard for lymphatic vessel invasion was the microscopic detection of cancer cells in the cavity of the lymphatic vessel by light microscopy.

All cases of recurrence had radiologic evidence or biopsy-proven progression of disease. Only the records of patients who died of disease were considered to be uncensored; the records of all patients who were alive at follow-up or who did not die of disease (or a related cause) were considered to be censored. Another 16 patients with non-tumor endometrial diseases undergoing routine endometrial biopsy were included as normal controls (NE).

## Methods

The antibody against LYVE-1 was purchased from R&D Systems (USA). Sections were dewaxed and antigen retrieval was carried out by microwaving in retrieval buffer (pH 6.0), three times for four minutes each. Slides were incubated in phosphate buffered saline (PBS) with 5% human serum for 5 minutes. Peroxidase was quenched with methanol and 3% H_2_O_2 _for 15 minutes. Then slides were incubated in antibodies to LYVE-1 (monoclonal mouse anti-human antibody 1.25 lg/ml), CD44 (DAKO, Denmark; mouse monoclonal antibody, 1:40). After incubation with the primary antibodies in PBS plus 5% fetal calf serum for 45 minutes and washing with PBS, sections were incubated with a secondary anti-mouse horseradish peroxidase conjugated antibody for 15 minutes and washed in PBS. The color was developed during a 15-minute incubation with diaminobenzidine (DAB) solution (DAKO) and sections were weakly counterstained with haematoxylin. Normal tissue sections from the small intestine were used as positive controls. PBS was substituted for primary antibody as the negative control.

### Determination of CD44 levels

Only nuclear staining of cells was considered positive for CD44. The neoplastic cells that were positively stained for CD44 antigen were counted in 10 high power fields (magnification factor of 400) for each tissue section and were scored semi-quantitatively as <10%, between 10% and 70%, or >70% cells positive [10].

### Lymphatic vessel counting

Lymphatic vessel counting was performed in the intratumoral, peritumoral, and normal tissues. As described by Ohno and Weidner [11,12], the areas of highest vascularization were chosen at low power (×100) and vessels were counted in three high power (×200) fields. The lymphatic vessel density (LVD) was the mean of the vessel counts obtained in these three fields.

### Data analysis

Data are given as mean ± SD. A one-way ANOVA test was used to evaluate the association between I-LVD or P-LVD and clinicopathological parameters. Survival curves were plotted using the method of Kaplan-Meier and compared using the log-rank test by dividing the two groups by their LVD at the mean value to give two similar-sized groups. A multivariate model using Cox stepwise regression analysis was used to assess the effect of tumor variables on survival. Differences were considered statistically significant for P values less than 0.05. Statistical analysis was done using SPSS statistical software (SPSS version 16.0).

## Results

### Clinicopathological characterization

The median age of the 102 patients at the time of diagnosis was 52.9 years (range 42-62 years). Forty-seven (46.1%) patients were diagnosed in FIGO stage I and twenty-six (25.5%) patients were diagnosed in FIGO stage II, whereas 29 (28.4%) were diagnosed in FIGO stage III. Seventy-six patients had an endometrioid adenocarcinoma (74.5%), 6 (6%) had adenoacanthoma carcinoma, 17 (16.6%) had papillary serous, and 3 (2.9%) had clear cell carcinoma. Lymph node sampling or dissection was generally performed in patients with deep myometrial invasion and/or high-grade or aggressive histological features. Pelvic and/or para-aortic lymph node sampling was performed for 59 patients (57.8%) and 27 of these (26.5%) had lymph node metastasis. Patient characteristics are outlined in Table 1. Of the 102 patients analyzed, 38 patients (37.3%) received radiation therapy and 18 (17.6%) denied a recommended radiation therapy. Thirty-five patients (34.3%) received an anti-hormone treatment. During the follow-up interval, 27 of 102 (26.5%) patients died. Tumor recurrence and metastasis were observed in 26 patients (25.5%) and 21 patients (20.6%) died of the tumor-related disease. The other 6 patients died of causes other than endometrial cancer.

**Table 1:** Relationship Between P-LVD or I-LVD and Clinical or Pathological variables in endometrial carcinoma

	N	P-LVD	P-value	I-LVD	P-value
Histologic subtype					
Endometrioid	76	20.62 ± 4.04		4.43 ± 1.11	
Adenoacanthoma	6	18.28 ± 4.40	0.540	4.08 ± 1.85	0.767
Papillary serous	17	19.71 ± 4.79		4.07 ± 1.27	
Clear cell	3	19.67 ± 7.54		4.00 ± 0.47	
Stage					
I	47	20.30 ± 3.92	0.016	4.25 ± 1.28	0.361
II	26	18.49 ± 4.93		4.15 ± 1.27	
III	29	21.79 ± 3.51		4.60 ± 0.83	
Grade					
Well differentiated	39	20.90 ± 4.57	0.576	4.59 ± 1.09	0.282
Moderately differentiated	32	19.89 ± 4.29		4.04 ± 1.34	
Poorly differentiated	31	20.00 ± 3.78		4.42 ± 0.96	
Myometrial invasion					
≥1/2	56	19.91 ± 4.67	0.543	4.45 ± 1.03	0.415
>1/2	46	20.60 ± 4.08		4.21 ± 1.31	
Nodal status					
Positive	27	22.08 ± 3.60	0.011	4.76 ± 0.87	0.074
Negative	75	19.62 ± 4.28		4.19 ± 1.23	
CD44 expression					
<10%	13	16.93 ± 1.84	0.000	3.84 ± 2.19	0.049
10%-70%	55	18.89 ± 3.53		4.14 ± 1.06	
>70%	44	23.18 ± 3.96		4.84 ± 0.97	
LVSI					
Positive	15	25.82 ± 2.79	0.000	4.86 ± 1.22	0.219
Negative	97	19.41 ± 3.73		4.29 ± 1.14	
Age					
≤55	54	20.00 ± 4.04	0.543	4.42 ± 1.06	0.682
>55	58	20.54 ± 4.40		4.30 ± 1.23	

P-LVD, peritumoral lymphatic vessel density; I-LVD, intratumoral lymphatic vessel density.

### LYVE-1^+ ^lymphatics in endometrial carcinoma

**Figure 1:**
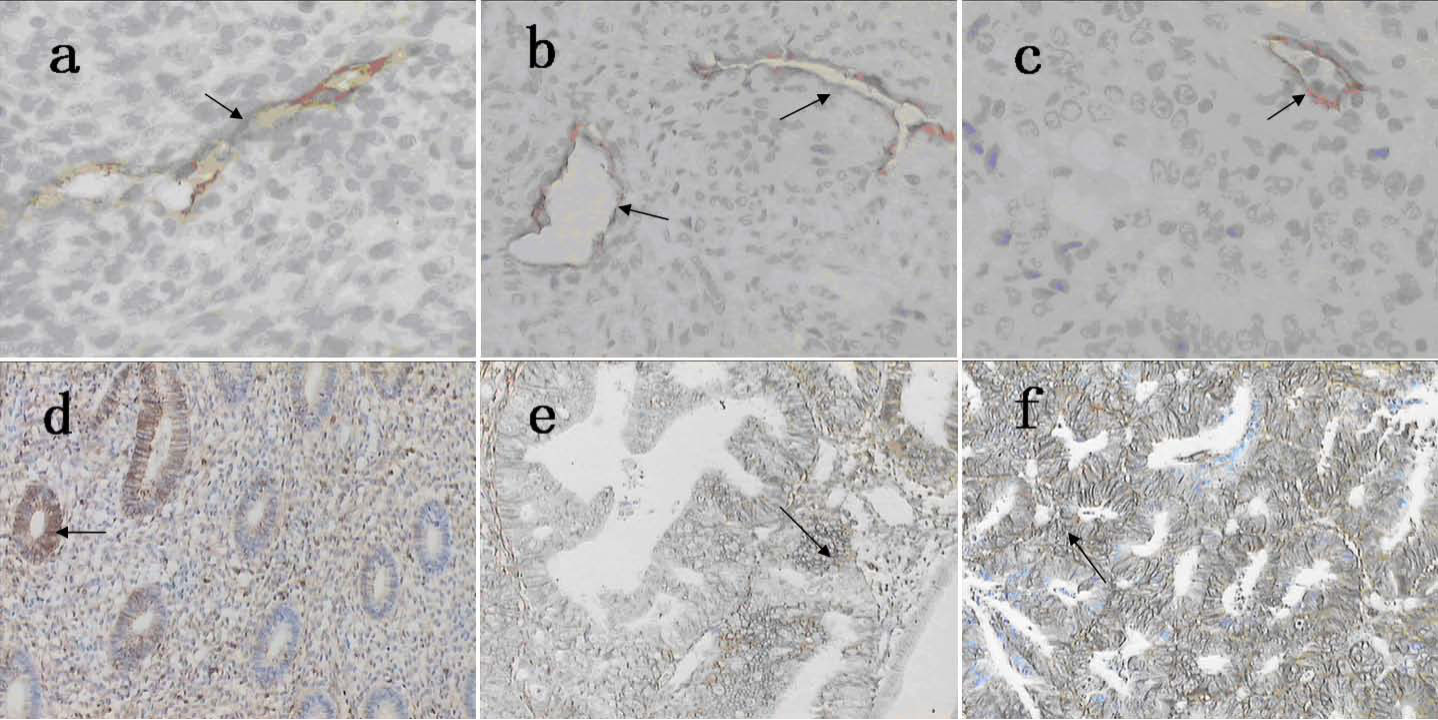
**a LYVE-1, normal endometrial tissue (NE), 200×**. **b **LYVE-1, periphery regions in endometrial carcinoma (EC) tissue, 200×. **c **LYVE-1, intratumoral regions in endometrial carcinoma (EC) tissue, 200×. LYVE-1 expression was restricted to irregular shaped endothelial cells without a thick-walled structure. Number of lymphatic vessels was much higher in periphery tumour than intratumoral regions (**b and c**). d The staining of CD44, normal endometrial tissue (NE), 200×. e CD44, in endometrial carcinoma (EC) tissue without LYVE1+ lymphatic vessels, 200×. f CD44, in endometrial carcinoma (EC) tissue with LYVE1+lymphatic vessels, 200×. The staining of CD44 was stronger in endometrial carcinoma (EC) tissue with LYVE1+lymphatic vessels than without LYVE1+ lymphatic vessels (e and f).

Lymphatic vessels were observed in EC (endometrial carcinoma) and NE (normal endometrium) specimens (Fig. 1a). LYVE-1^+ ^cells were predominantly found in the peripheral regions of tumor nests; staining was weak in intratumoral regions. LYVE1 staining in peripheral regions was interpreted as positive in 94 of the 102 (92.2%) samples. Within tumors, 64 (62.7%) of the 102 samples were positive. Inside the tumors, the small vessels with LYVE-1^+ ^endothelial cells were of irregular shape without a thick-walled structure (Fig. 1c). In contrast, prominently large, irregular morphology, thin-walled lymphatics were detected at the periphery of the tumor. Some small and depressed vessels were detected inside the carcinoma tissue (Fig. 1b and 1c). In the 94 LYVE1^+ ^samples, the mean density at the tumor periphery (P-LVD) was 20.30 ± 4.23 vessels/mm^2^. In intratumoral regions, the average density (I-LVD) was significantly lower: 4.35 ± 1.15 vessels/mm^2^. Both densities were significantly different from the density in normal control tissue (17.17 ± 2.13 vessels/mm^2^).

### Association of LVD with clinicopathological parameters

P-LVD was significantly correlated with lymph node metastasis, LVSI, tumor stage and CD44 expression in endometrial carcinoma (Table 1, P < 0.05). In cases with nodal metastasis, P-LVD was 22.08 ± 3.60 vessels/mm^2 ^(P = 0.011, compared with P-LVD in negative nodal metastasis cases 19.62 ± 4.28 vessels/mm^2^). In addition, P-LVD was significantly associated lymphatic vessel invasion (P < 0.01). In 102 endometrial carcinoma cases, CD44 was immunohistochemically detected in 94 cases (92.2%). P-LVD in patients with strong nuclear CD44 staining of cells (>70%) was 23.18. ± 3.96 vessels/mm^2^, P-LVD in samples with intermediate CD44 expression (10-70%) was 18.89. ± 3.53 vessels/mm^2^, and in those with weak CD44 expression (10%) P-LVD was 16.93. ± 1.84 vessels/mm^2 ^(P < 0.01). The staining of CD44 was stronger in endometrial carcinoma tissue with LYVE1^+ ^lymphatic vessels than without LYVE1^+ ^lymphatic vessels (Fig. 1e and 1f). In multivariate analysis, P-LVD was independently correlated with LVSI, tumor stage and CD44 expression (P = 0.561).

No significant difference in I-LVD was observed in samples from patients with and without nodal metastasis (P = 0.074). I-LVD was similar between the patients with LVSI and those without LVSI (P = 0.219). I-LVD was significantly correlated with CD44 staining, however (Table 1, P = 0.049). The differences in I-LVD between different tumor stages had no statistical significance. The tumors associated with increased P-LVD or I-LVD tended to have higher CD44 expression, higher tumor stage, and nodal metastasis. No significant association was found between P-LVD or I-LVD and the depth of myometrial invasion, histological classification, or grade. There was no correlation between LVD and patient age (Table 1).

### Survival analysis

**Figure 2:**
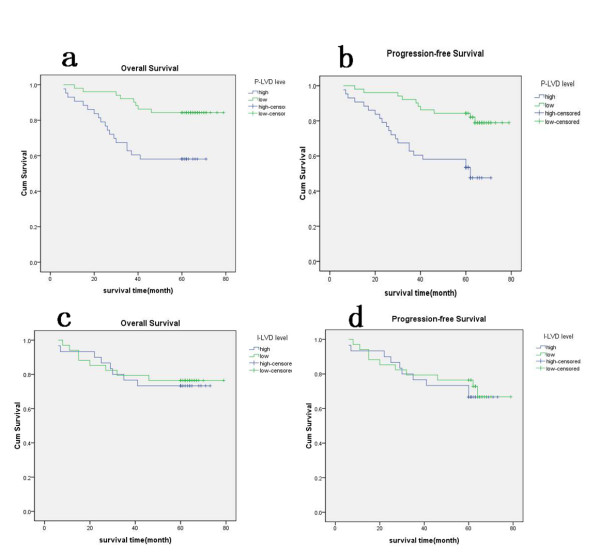
**Survival analysis of increased P-LVD and I-LVD in endometrial carcinoma**. a Overall-survival curve stratified for increased versus decreased P-LVD (P < 0.01). b Progression-free survival curve stratified for increased versus decreased P-LVD (P < 0.01). c Overall-survival curve stratified for increased versus decreased I-LVD (P > 0.05). d Progression-free survival curve stratified for increased versus decreased I-LVD (P > 0.05).

The mean follow-up time was 54.2 months (range 5-79 months). During the follow-up interval, 27 of 102 (26.5%) patients died of disease. Twenty patients developed pelvic local recurrence (among them, 15 patients were presented strong CD44 staining). Six patients had distant dissemination (including three cases of relapse in the lung, one case of liver metastasis, one case multiple metastasis, and one case of para-aortic nodal metastasis). The 5-year survival rate was 73.5% and progression-free survival rate was 68.6%. To analyze the correlation between I-LVD or P-LVD and survival, patients were divided into two groups: one group with P-LVD or I-LVD higher than mean and another group with P-LVD or I-LVD lower than the mean. In univariate analysis, increased P-LVD was associated with poorer overall survival (P < 0.01, Fig. 2a) and less likelihood of progression-free survival (P < 0.01, Fig. 2b). A significantly better survival rate and likelihood of progression-free survival was found in patients with lower P-LVD. In contrast, the presence of I-LVD was not associated with survival (P > 0.05, Fig. 2c and Fig. 2d). Other clinical parameters, including LVSI, FIGO stage, histologic subtype, and nodal status of endometrial cancer, significantly affected the survival rates in the evaluated patients, demonstrating the validity of the patient group enrolled in our study (P in log rank was <0.01 for both progression-free and overall survival). Cox regression analysis allowed us to construct a prognostic model containing three independent terms that were predictive of overall and progression-free survival: P-LVD, FIGO stage, and histologic subtype (P < 0.05). Nodal involvement and LVSI were not independent prognostic factors in a multivariate survival analysis (Table 2).

**Table 2:** Cox regression analysis determining the independent effect of prognostic variables on survival

varible	progression-free survival			overall survival		
	
	HR	95%CI	p-value	HR	95%CI	p-value
Histologic subtype	1.5	1.1-2.0	0.005	1.6	1.2-2.1	0.003
Stage	2.4	1.3-4.4	0.005	2.3	1.2-4.4	0.014
Nodal metastasis	1.8	0.6-5.4	0.309	1.5	0.5-5.0	0.487
P-LVD	0.2	0.1-0.6	0.003	0.3	0.1-0.8	0.019
LVSI	1.1	0.4-2.8	0.872	1.4	0.5-3.7	0.479

## Discussion

The study of lymphatics has been facilitated by the identification of the lymphatic marker LYVE-1 [13-17]. LYVE-1 is found in lymphatic endothelium and not in blood vascular endothelium except in the sinusoidal endothelium of liver and spleen where uptake and degradation of HA is known to occur [17]. There is controversy regarding lymphatics and lymphangiogenesis in the endometrium. There is a report that lymphatic vessels of endometrial adenocarcinomas were located both intra- and peri-tumorally and that vessel density was significantly higher than in normal basalis; moreover, a potential vascular-control feedback loop was indentified [18]. In contrast, two studies reported an absence of human endometrial lymphatics [19,20]. In this study, LYVE-1 positive vessels were identified as lymphatics in endometrial carcinoma tissue. The I-LVD of carcinoma tissue was significantly lower than that of normal tissue, whereas the P-LVD was higher. In our experiment, small, depressed lymph vessels were mostly observed within the carcinoma tissues and the peritumoral lymph vessels were large and dilated. Lymphatic vessels formed anastomoses and possessed frequent blind endings that were occasionally open. Tripp et al. [21] found inter-connected LYVE-1^+ ^vessels that went into the deeper dermis in skin. Similarly, a lymphatic network may exist in endometrial tumors in both the peripheral and intratumoral regions. This data contradicts a study by Kouk et al. [20] that did not find intratumoral lymph vessels or an intratumoral lymphatic network in endometrial carcinomas. It seems clear that lymphatic and vascular systems have numerous connections that allow disseminating cancer cells to pass rapidly from one system to another [22]. The formation of a lymphatic network within a tumor greatly facilitates tumor growth by draining waste products of metabolism and promoting lymphatic dissemination of tumor cells.

As a tumor grows, the lymphatics inside the tumor are compressed or destroyed and become difficult to detect by immunohistochemistry. This is consistent with our findings that the I-LVD of carcinoma tissues was significantly lower and the P-LVD was significantly higher than that of normal endometrium. Two recent studies in melanoma (using the anti-LYVE-1) confirmed the presence of focal areas exhibiting intratumoral lymphatic proliferation [23,24]. In addition, the presence of intratumoral lymphatics in laryngeal carcinoma may assist tumor spread to regional lymph nodes [25]. However, whether intratumoral lymphangiogenesis is necessary for or enhances lymphatic metastasis is still unclear.

In our study, the P-LVD had the highest correlation with patient survival of the parameter evaluated. The peritumoral lymphatics are likely important for dissemination of endometrial carcinoma cells. In support of this hypothesis, our study demonstrated that high P-LVDs were associated with LVSI, lymph nodes metastasis, and poor likelihood of survival. The results showed that LYVE1 is a useful prognostic tool. The lymphangiogenesis and location of lymphatic vessels relative to a primary tumor may be a determinant of metastatic spread. As reviewed by Achen [26], the VEGF-C/VEGF-D/VEGFR-3 axis was the best validated signaling system implicated in promoting lymphangiogenesis in solid tumors and the metastatic spread of tumor cells to lymph nodes. These growth factors were also likely candidates for driving lymph node lymphangiogenesis and appear to promote metastatic spread to sentinel lymph nodes and perhaps to more distant sites. Metastasis may also depend on the position of the primary tumor relative to the lymphatic network. P-LVD may be a determinant of metastatic spread as studies in animal models indicate that peritumoral lymphatics are capable of draining fluid and cells from a tumor whereas intratumoral lymphatics are nonfunctional [27]. It may be that an extensive peritumoral lymphatic network produces high concentrations of VEGF-C and other growth factors that promote angiogenesis and lymphangiogenesis of distant metastases, enhancing further dissemination and growth of cancer. The peritumoral lymphatics may be a target for inhibition of metastasis. Clinically, the patients with high P-LVD should be considered for early chemotherapy and should be re-evaluated often.

In addition to lymphangiogenesis, the interaction between epithelial tumor cells and their surrounding stroma is important in tumor progression and metastasis. As a transmembrane receptor interacting with stromal ECM, CD44 plays an important role in the process through binding to HA. It was reported that with hyperplasia, increasing atypia and adenocarcinoma [8], the levels of stromal HA, glandular CD44v6, and glandular and stromal CD44s increase. CD44 facilitates breast cancer progression through alterations of tumor cell adhesion characteristics [28,29]. CD44 is a mesenchymal stem cell (MSC) marker; the rare epithelial progenitors and MSCs are likely responsible for regenerative capacity that plays a critical role in the development of endometriosis and endometrial cancer. Several versions of CD44v6-specific antibodies have been tested in clinical trials and preliminary results are promising [30]. It may provide a readily available source of MSCs for cell-based therapies [31]. The CD44 gene contains at least 20 exons, 10 of which can be alternatively spliced to form a variety of isoforms. Exons 6-15 can be alternatively spliced to generate many CD44 variants (CD44v) mainly expressed on epithelial cells [32]. CD44v8-10 is overexpressed in various malignant tumors and is considered to be associated with the presence of tumor or with tumor progression. An elevated CD44v8-10-to-standard CD44 ratio in urinary samples may serve as a novel prognostic predictor in patients with urothelial cancer [33]. CD44v8-v10 can be produced from epithelial cells, but no studies have addressed CD44v8-v10 expression on lymphatics until now. Here we found that CD44 had prognostic significance. Both increased P-LVD and I-LVD were correlated with stronger CD44 staining. Although patients with high levels of CD44 staining did not have significantly reduced overall or progression-free survival in our study, 15 of the 20 patients who developed pelvic local recurrence had strong CD44 staining. Alterations of CD44 expression occurred during local pelvic recurrence. In contrast, the alterations of LYVE1 were associated with the dissemination of the disease. The main physiological functions of LYVE-1 include serving as a receptor for HA and facilitating the transport and metabolism of HA in the extracellular matrix. It has also been shown that LYVE-1 is involved in adherence of tumor cells [9]. LYVE-1 may promote tumor lymph node metastasis and lymphatic invasion. Based on their similar adhesive functions, LYVE1 and CD44 may both contribute to metastatic spread of cancer cells since HA is more abundant in the surrounding stroma than in the tumor itself. From a clinical point of view, the analysis of LYVE1 and CD44 expression in endometrial carcinomas may help to identify patients with poor prognosis who may benefit from adjuvant therapies.

## Conclusions

P-LVD was significantly correlated with lymph node metastases, lymph vascular space invasion (LVSI), tumor stage, and CD44 expression in endometrial carcinoma samples. The P-LVD was an independent risk factor for progression-free survival and overall survival. Therefore, P-LVD may serve as a prognostic factor in endometrial carcinoma and the peritumoral lymphatics might play an important role in lymphatic vessel metastasis.

## Competing interests

The authors declare that they have no competing interests.

## Authors' contributions

YG was the guarantor of integrity of the entire study and designed the experiment, carried out the immunohistochemical method studies and drafted the manuscript. ZL participated in literature research, data analysis and manuscript editing. FG participated in the design of the study and performed the statistical analysis. X.Y-M carried out the immunohistochemical method studies and participated in manuscript preparation. All authors read and approved the final manuscript.

## Pre-publication history

The pre-publication history for this paper can be accessed here:

http://www.biomedcentral.com/1471-2407/10/131/prepub
